# Fabrication of Functionally Graded Diamond/Al Composites by Liquid–Solid Separation Technology

**DOI:** 10.3390/ma14123205

**Published:** 2021-06-10

**Authors:** Hongyu Zhou, Yaqiang Li, Huimin Wang, Minrui Ran, Zhi Tong, Weidong Zhang, Junyou Liu, Wenyue Zheng

**Affiliations:** 1 National Center for Materials Service Safety, University of Science and Technology Beijing, Beijing 100083, China; hyzhou@ustb.edu.cn (H.Z.); wanghuimin@ustb.edu.cn (H.W.); 18600179244@163.com (M.R.); tongzhi199308@163.com (Z.T.); zwd@ustb.edu.cn (W.Z.); 2 Institute for Advanced Materials and Technology, University of Science and Technology Beijing, Beijing 100083, China; yqli0677@163.com; 3 School of Materials Science and Engineering, University of Science and Technology Beijing, Beijing 100083, China

**Keywords:** diamond/Al composites, near-net shape forming, functionally graded composites, interface, coefficient of thermal expansion, liquid–solid separation

## Abstract

The electronic packaging shell, the necessary material for hermetic packaging of large microelectronic device chips, is made by mechanical processing of a uniform block. However, the property variety requirements at different positions of the shell due to the performance have not been solved. An independently developed liquid–solid separation technology is applied to fabricate the diamond/Al composites with a graded distribution of diamond particles. The diamond content decreases along a gradient from the bottom of the shell, which houses the chips, to the top of the shell wall, which is welded with the cover plate. The bottom of the shell has a thermal conductivity (TC) of 169 W/mK, coefficient of thermal expansion (CTE) of 11.0 × 10^−6^/K, bending strength of 88 MPa, and diamond content of 48 vol.%. The top of the shell has a TC of 108 W/mK, CTE of 19.3 × 10^−6^/K, bending strength of 175 MPa, and diamond content of 15 vol.%, which solves the special requirements of different parts of the shell and helps to improve the thermal stability of packaging components. Moreover, the interfacial characteristics are also investigated. This work provides a promising approach for the preparation of packaging shells by near-net shape forming.

## 1. Introduction

**Figure 1:**
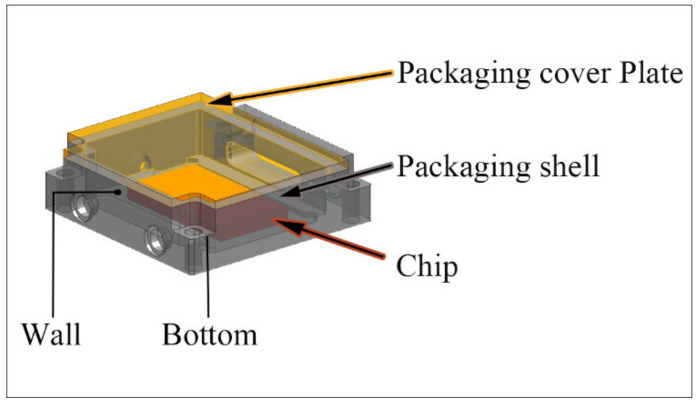
Diagram of a packaging assembly.

The continuous development of microelectronic systems has resulted in the highly compact size of electronic devices and the dramatic increase in power density, which requires advanced thermal management material, housing the chip, to ensure efficient heat dissipation [1,2]. Diamond/Al composites have been studied extensively due to their excellent thermal conductivity (TC), tailorable coefficient of thermal expansion (CTE), and low density [3,4]. However, unlike heat sink substrates, the packaging shell has different performance requirements for different parts, as shown in Figure 1. The part of the housing chip should have a low CTE to match the chip, while the part of the top packaging wall should have a high solderability to ensure brazing with the packaging cover plate [5]. At present, the packaging shell is made by mechanical processing of a uniform block, but the different performance requirements have not been solved.

Compared with the single or monotonous material, the functionally graded materials (FGMs) possess properties that enable the reduction in the thermal stress concentration [6]. The FGMs with high diamond content at the bottom of the shell and low diamond content at the top of the wall also solve the above problems, improving the stability of the packaging components. Furthermore, the FGMs can resist forceful high-temperature grading while maintaining structural integrity, eliminating/reducing interface problems, thermal stress concentration, residual stress, etc. [7]. Nonetheless, in the mainstream diamond/Al composites preparation methods, such as pressureless infiltration [8], gas pressure infiltration [9], squeeze casting [10], vacuum hot pressing [11], powder metallurgy [12], and spark plasma sintering [13], the near-net-shape graded composites have not been reported. In addition, the interface products produced during the preparation process, such as Al_4_C_3_, are also the crucial factor affecting the mechanical properties and thermal conductivity of the composites [14,15]. The formation of Al_4_C_3_ deteriorates the properties of the diamond/Al composites by reducing interfacial thermal conductivity [16], mechanical properties [17], and stability when serviced in wet environments [18]. The coating on the surface of diamond particles is considered an effective way to avoid the formation of Al_4_C_3_ [11,19]. However, Li [9] and Xin [14] have found that Al_4_C_3_ is still formed at the interface due to the high temperature and long holding time of the preparation process, even if the surface of diamond particles has a complete metal coating. Therefore, under the premise of avoiding the production of Al_4_C_3_ at the interface, the development of near-net shape forming technology of the diamond/Al composite packaging shell is of great significance for promoting the application of a new generation of thermal management materials.

In recent years, a liquid–solid separation (LSS) technology uses a thixoforming process of semi-solid metals, including a liquid and solid two-phase that flows under pressure, allowing a high-volume fraction of the solid phase to be retained [20]. This compact route and low-cost method to the preparation of diamond/Al composites with a suitable sintering temperature and time has been confirmed without the formation of Al_4_C_3_ at the interface between diamond and Al [21]. At present, only diamond/Al composites packaging substrates with regular shapes can be fabricated by the LSS process. In this work, we further utilized the phenomenon that the fluidity of diamond particles is different from that of liquid Al under pressure, optimized the LSS mold system, and finally prepared the diamond/ Al composites with a graded distribution of diamond particles. It is significant in guiding the industrialization and popularization of these types of materials, such as diamond/Cu composites and diamond/Ag composites, by analyzing the graded distribution of the microstructure and properties of diamond/Al composites.

## 2. Materials and Methods

### 2.1. Materials

**Figure 2:**
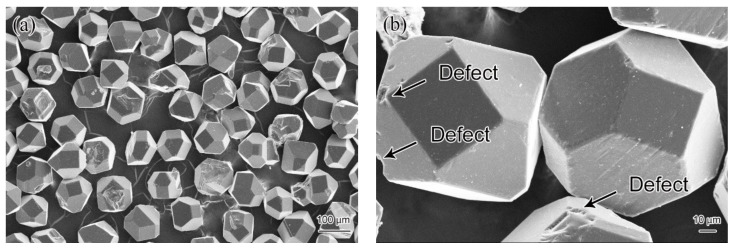
Microscopic morphology of the diamond particles: (**a**) low magnification and (**b**) high magnification of SEM images.

As the matrix material, commercial Al (99.81 wt.%) powders with an average size of about 37 µm were provided by Zhengzhou Aerospace Aluminum Co., Ltd. in Zhengzhou, China. As the reinforcement material, MBD-4-type synthetic diamond particles with an average size of about 106 µm were manufactured by Henan Huanghe Whirlwind Co., Ltd. in Xuchang, China. Figure 2a displays the morphology of the diamond particles at low magnification. The size of diamond particles is relatively uniform while some of them are broken, showing an incomplete shape. It can also be seen from the high magnification image (Figure 2b) that there are only a few visible defects on the diamond surface (shown by the black arrow).

### 2.2. Preparation of FGMs

**Figure 3:**
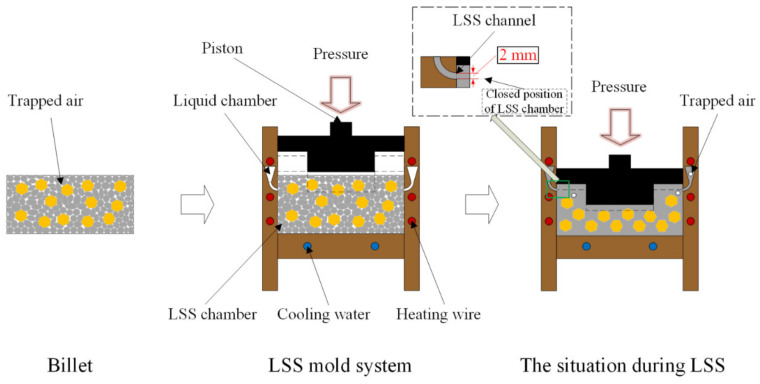
Schematic diagram of the LSS technology.

The diamond particles and Al powders were mechanically mixed with the volume ratio of 1:4 via 3D Turbula (Model T2C, Glen Mills, Germany) mixing for 8 h. The mechanically mixed material was compressed into a billet with a size of 48 mm × 38 mm × 7.5 mm by a hydraulic machine (YQ28-100, Wodda, China) at the pressure of 300 MPa for 1 min. Figure 3 is a schematic diagram of the LSS technology. As shown in Figure 3, the billet was transferred to a custom-made LSS mold system and heated to a liquid–solid mixed-melting state. The sintering parameters were 683 °C with a heating rate of 20 °C·min^−1^. To complete the LSS process, the liquid–solid mixed melt slurry was squeezed with a special shape piston at the pressure of 60 MPa to fill the LSS chamber to form the shell shape. The extruded melted hot Al entered the liquid chamber through the 2 mm LSS channel while the diamond particles were completely retained in the LSS chamber. At that time, the piston moved to the closed position of the LSS chamber to ensure the high relative density of the packaging shell. The remaining liquid–solid mixed slurry solidified layer by layer from bottom to top under the water-cooling system, and the pressure was held for 240 s during the solidification process.

The previous experiments [20,21] have fully proved that the 2 mm LSS channel can completely retain the diamond particles with a diameter of 106 μm in the LSS chamber. Composites with different volume fractions are precisely prepared by changing the volume of the LSS chamber. There is no horizontal component in the center of the bottom of the composite, and the diamond particles in this part are completely concentrated. After calculation, the thickness of the bottom at the center part of the packaging shell was 3 mm when the diamond content reached 50 vol.%. The specific size parameters of the packaging shell are described in the results and discussion section.

### 2.3. Characterization

The diamond/Al composite specimens made with the above method were machined by a laser cutting machine and a diamond wheel grinder. The microstructure of the diamond/Al composites was characterized by an EVO-18 scanning electron microscope (SEM, Zeiss, Germany). The bending strength was tested using an RGM-3010 electronic universal testing machine (Reger, China) for the sample with dimensions of 3 mm × 4 mm × 25 mm. The density (*ρ*) of the fabricated composite was tested with a GH-120 E automatic density measuring instrument (Xiamen Qunlong, China) for the sample with dimensions of φ 10 mm × 3 mm. The distributions of main elements between Al and diamond particles were detected using a JXA-8230 electron microprobe analysis (EMPA, JEOL, Japan). The phase composition of the diamond/Al composites was confirmed by Advance D8 X-ray diffractometer (XRD, Bruker, Germany) at 40 kV and 35 mA, using Cu Ka radiation. A total of 2θ scans were performed between 20° and ~80° with a scan speed of 4 °/min. The thermal diffusivity (α) of the diamond/Al composites was tested by an LFA 427 laser flash thermophysical machine (NETZSCH, Germany) at room temperature with a sample size of φ 10 mm × 3 mm. The specific heat capacity (C_p_) was calculated theoretically according to the rule of mixture (ROM). The TC (λ) can be obtained by formula: λ = ρ × α × C_p_. The CTE of the diamond/Al composites was measured by a DIL 402C differential dilatometer (NETZSCH, Germany) over a temperature range of room temperature to 200 °C at a heating rate of 5 °C/min with a sample size of 3 mm × 4 mm × 25 mm. For all testing, three specimens were performed for each measurement to acquire the average value.

## 3. Results and Discussion

### 3.1. Microstructure of FGMs

**Figure 4:**
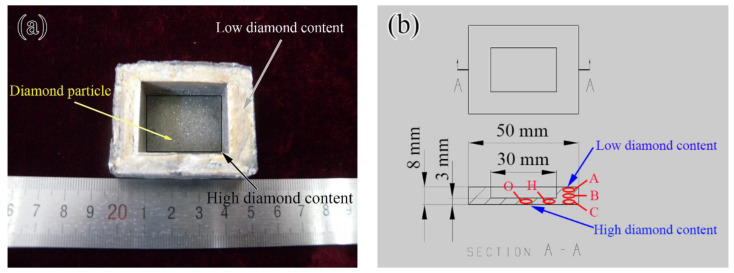
Diamond/Al graded composite fabricated by LSS process: (**a**) macroscopic view, (**b**) sampling positions. Sampling positions of “O”, “H”, “C”, “B”, and “A” have the coordinates of (0,0), (10,0), (20,0), (20,3.25), and (20,6.5), respectively. “O” is positioned at the center of the shell bottom.

Figure 4a shows the macroscopic view of a diamond/Al graded composite fabricated by the LSS process. The synthetic single-crystal diamond particles are generally pale yellow under the naked eye. It can be seen that the Al matrix is inlaid with diamond particles with a yellow shine at the bottom of the shell. In contrast, no diamond particles were observed, only metallic sheen, at the top of the shell wall. The macroscopic view of the shell indicates that the diamond particles present the graded distribution in the composite. Figure 4b shows the dimensions of the composite shell and the sampling positions. The outer dimension of the shell is 40 mm × 50 mm × 8 mm, and the central cavity dimension of the shell is 20 mm × 30 mm × 5 mm. As shown in Figure 4b, the sampling positions of “O”, “H”, “C”, “B”, and “A” have the coordinates of (0,0), (10,0), (20,0), (20,3.25), and (20,6.5), respectively. “O” is positioned at the center of the shell bottom.

**Figure 5:**
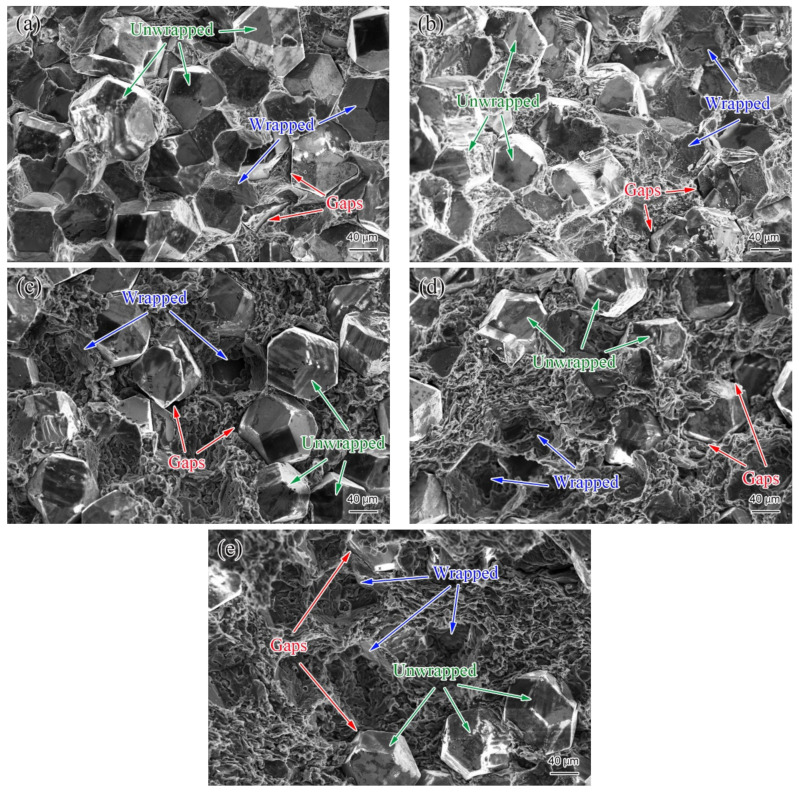
Microstructural distribution of the diamond/Al graded composite: (**a**) point “O”, (**b**) point “H”, (**c**) point “C”, (**d**) point “B” and (**e**) point “A”. Blue arrows indicate diamond particles are integrally wrapped by metal Al, green arrows indicate diamond particles are not wrapped by Al and red arrows indicate gap area between diamond and Al.

Figure 5 shows the three-point bending fracture morphology of the diamond/Al graded composite at different positions. The fracture morphology of the composite varies with the sampling positions. Along the horizontal direction of the bottom of the shell, i.e., point “O” (Figure 5a) → point “H” (Figure 5b) → point “C” (Figure 5c), the distance between adjacent diamond particles increases gradually, i.e., the Al matrix filling between the diamond particles increases gradually from the center to the edge. This indicates that in this direction, the content of diamond particles of the composite decreases successively. Additionally, from the bottom to the top in the wall of the shell, i.e., point “C” → point “B” (Figure 5d) → point “A” (Figure 5e), the content of diamond particles also decreases successively. To sum up, from the center of the bottom of the shell to the top of the wall, the diamond content decreases gradually, which is consistent with the macroscopic morphology of the composite in Figure 4a. The graded distribution of the reinforcement phase perfectly solves the special requirements of different parts of the packaging shell for performance and helps to improve the thermal stability of packaging components [5]. The blue arrow in Figure 5 shows that some diamond particles are integrally wrapped by metal Al, indicating that the interfacial bonding strength between diamond and Al is high in this area. The dimples are also observed in the Al matrix, indicating a large amount of plastic deformation in the fracture zone, which has a high level of toughness [22]. However, some diamond particles are not wrapped by Al due to weak adhesion of the interface, as shown by the green arrow in Figure 5. The red arrow in Figure 5 directly observed the morphological characteristics of interface debonding, which also verified that the interface bonding strength is not high. In the process of plastic deformation, the dislocations multiply, and form micropores when the dislocations reach a certain density, leading to disconnection of the interfaces of the matrix and reinforcement phase [23]. The results show that the interfacial bond between diamond and Al needs to be further strengthened to improve the properties of the composites.

**Figure 6:**
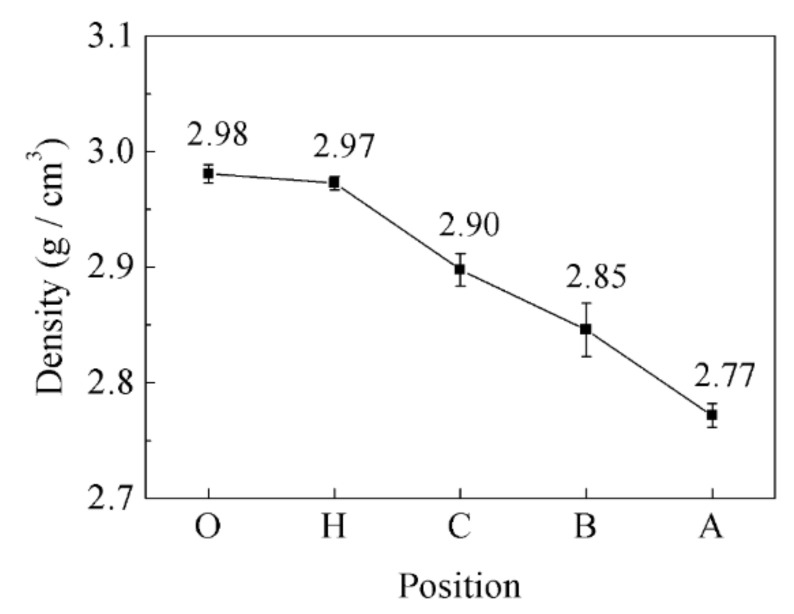
Variation of the density of the diamond/Al graded composites at different sampling positions.

The density of the diamond/Al graded composites at different sampling locations is shown in Figure 6. The density of the samples at point “O”, point “H”, point “C”, point “B”, and point “A” are 2.98 g/cm^3^, 2.97 g/cm^3^, 2.90 g/cm^3^, 2.85 g/cm^3^, and 2.77 g/cm^3^, respectively. From the center of the bottom of the shell to the top of the wall, the density of the diamond/Al graded composites decreases gradually, which corresponds to the microstructure of the composites (Figure 5). The content of the diamond particles of diamond/Al graded composites is described in detail in Section 3.4. The properties of the composites are optimized by FGMs whose microstructure is distributed along the specified direction [24].

### 3.2. Interfacial Characteristics of FGMs

**Figure 7:**
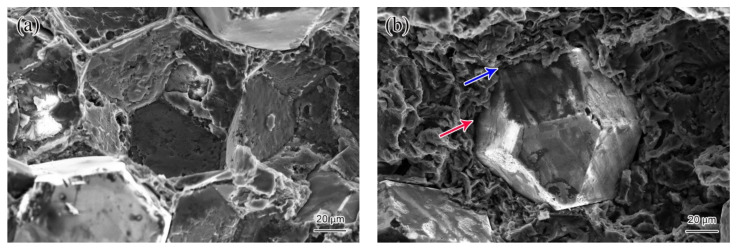
Interface morphology of the diamond/Al graded composites. (**a**) Diffusion-bonded, (**b**) partially diffusion-bonded. Red arrow indicates interface is non-bonded and blue arrow indicates interface is diffuse bond.

**Figure 8:**
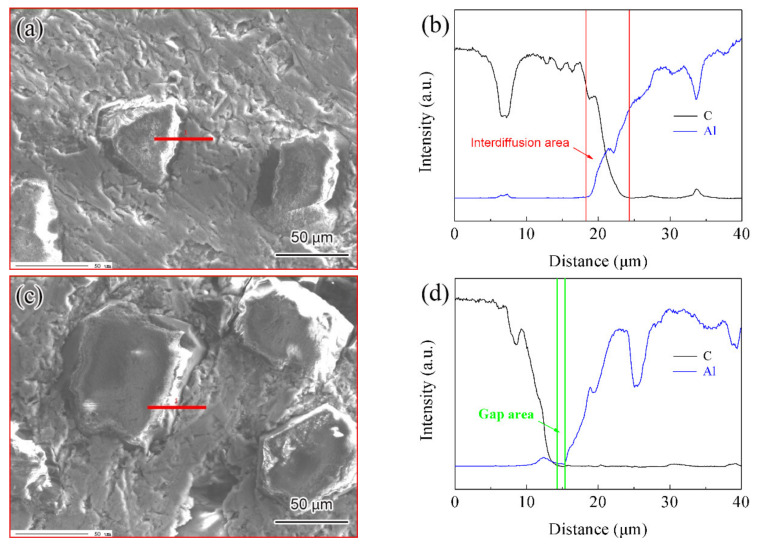
Element distribution of the interface of the diamond/Al graded composites: (**a**,**c**) SEM image, (**b**,**d**) EMPA map.

Figure 7 shows the interface morphology of the diamond/Al graded composites. The well wrapped diamond particle by metal Al is shown in Figure 7a, which indicates the strong interface connection between diamond particles and Al matrix. The EMPA results also confirm that the mutual diffusion region is formed in the region where the Al signal intensity increases sharply while the C signal intensity decreases sharply, as shown in Figure 8a,b. The width of the mutual diffusion region is about 8 µm, indicating that the interfacial area between diamond and Al is metallurgical bonding, which improves the properties of the composites [25]. However, Figure 7b shows that the interface bonding between diamond particles and Al is partially diffusion-bonded due to non-wetting and solidification shrinkage. The area indicated by the red arrow is non-bonded, while the area indicated by the blue arrow is diffuse bond. The gap area across the interface, in which there is no contact between diamond particles and Al, is shown in Figure 8c,d. The distribution of elements at the interface shows that the gap area is about 2 µm. During the solidification process, the voids and pores of diamond/Al composites reach more than 2 vol.% due to the difference of CTE between Al and diamond particles [26], which also reduces the interface bonding of composites. The interface of partial diffusion bonding is not enough to form the ligament connecting diamond particles and Al, and reduces the properties of the composites [27].

**Figure 9:**
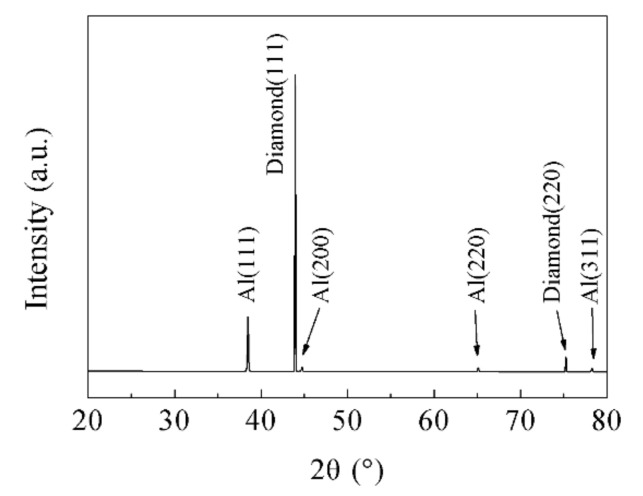
XRD pattern of the diamond/Al graded composites.

It is noteworthy that no selective bonding of Al on the {100} facets of the diamond is found in Figure 5 and Figure 7. Selective bonding is the microscopic manifestation of the formation of Al_4_C_3_ [28], which demonstrates that the sintering temperature and time of the LSS process are reasonable. The XRD pattern of the diamond/Al graded composites (Figure 9) manifests the diffracted peaks of Al and diamond, while the diffracted peaks corresponding to Al_4_C_3_ are not detected. No Al_4_C_3_ phase was found at the scanning speed of 0.25°/min for the diamond/Al composites packaging substrate prepared by the LSS process [21].

### 3.3. Properties of FGMs

**Figure 10:**
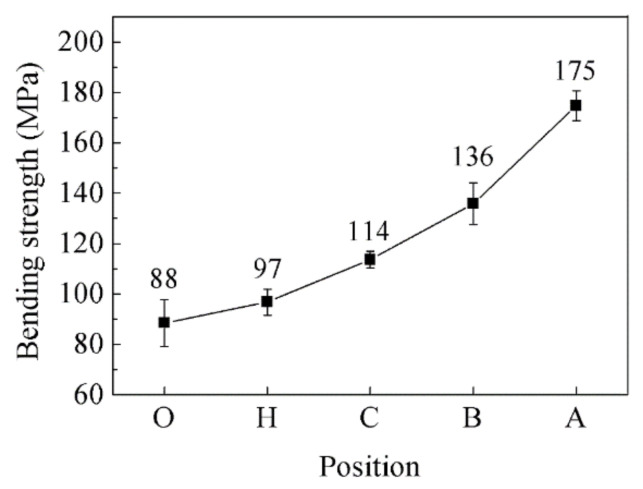
Bending strength of the diamond/Al graded composites at different sampling positions.

Table 1 shows the thermophysical property and bending strength of the diamond/Al graded composites at different sampling positions. With the decrease in diamond content, the bending strength are 88.47 MPa, 96.77 MPa, 113.60 MPa, 135.86 MPa, and 174.72 MPa, respectively. The bending strength increases with decreasing content of diamond particles in the Al matrix, as shown in Figure 10. The decrease in the content of diamond particles is beneficial to increase the bending strength of diamond/Al composites [11,29].

**Figure 11:**
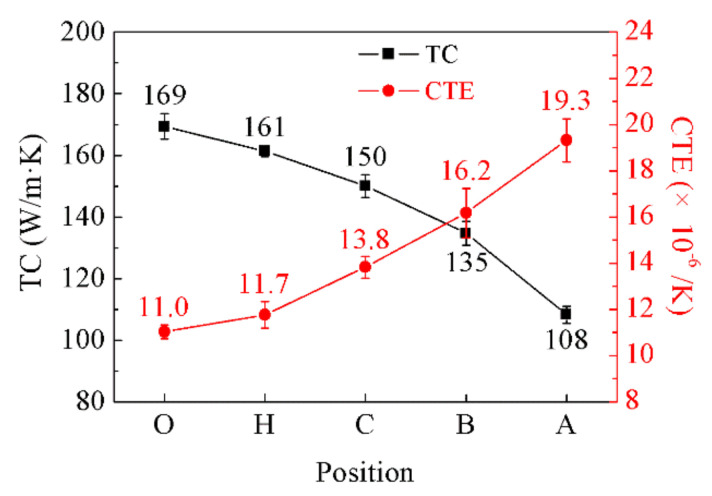
TC and CTE of the diamond/Al graded composites at different sampling positions.

The TC of the samples at point “O”, point “H”, point “C”, point “B”, and point “A” are 169.36 W/m·K, 161.38 W/m·K, 150.02 W/m·K, 135.65 W/m·K, and 108.25 W/m·K, respectively. Additionally, the CTE of the samples are 11.0 × 10^−6^/K, 11.8 × 10^−6^/K, 13.8 × 10^−6^/K, 16.2 × 10^−6^/K, and 19.3 × 10^−6^/K, respectively. Figure 11 demonstrates the variation of TC and CTE of the diamond/Al graded composites at different sampling positions. With the decrease in diamond content, the TC of the composites decreased, while the CTE increased. The sample at point “O” has the lowest CTE (11.0 × 10^−6^/K) and the highest TC (169 W/m·K), such performance ensures that the CTE of the composites is matched with the CTE of the chip, and dissipates the heat generated by the chip calculation in a timely and effective manner. The sample at point “A” has the highest CTE (19.3 × 10^−6^/K), which is close to the CTE of the metal cover plate, ensuring good weldability. The graded distribution of the diamond particles perfectly solves the special requirements of different parts of the packaging shell for performance and helps to improve the thermal stability of packaging components [5]. In addition, the gradual change in the structure of the packaging shell, reducing the thermal stress and residual stress, helps to improve the thermal stability of the packaging components [7]. The numerical simulation studies of heat transfer behaviors also confirmed that heat gradually decreases along the graded direction of FGMs, reducing the thermal stress caused by the mismatch in CTE between different layers [6].

### 3.4. Diamond Distribution of FGMs

The Kerner model [30], which takes the shear effect into account at the boundary between reinforcement and matrix, has been widely used in the theoretical calculation of CTE of the composites [31]. The Kerner model can be expressed in Equation (1).

(1) $$\alpha_{c}{=\alpha }_{m}V_{m}{+\alpha }_{d}V_{d}{+V}_{d}V_{m}(\alpha_{d}\;-\;\alpha_{m})\;\times \;\frac{K_{d}-K_{m}}{V_{m}K_{m}+V_{d}K_{d}+\frac{3K_{d}K_{m}}{4G_{m}}}$$

where $\alpha_{c}$ is the CTE of the composite, $\alpha_{d}$ the CTE of the reinforcement phase, $\alpha_{m}$ the CTE of the matrix, $V_{d}$ the content of the reinforcement phase in the composites, $V_{m}$ the content of the matrix in the composites, $K_{d}$ the bulk modulus of the reinforcement phase, $K_{m}$ the bulk modulus of the matrix, and $G_{m}$ the shear modulus of the matrix. Table 2 shows the parameters involved in the Kerner model [32].

**Figure 12:**
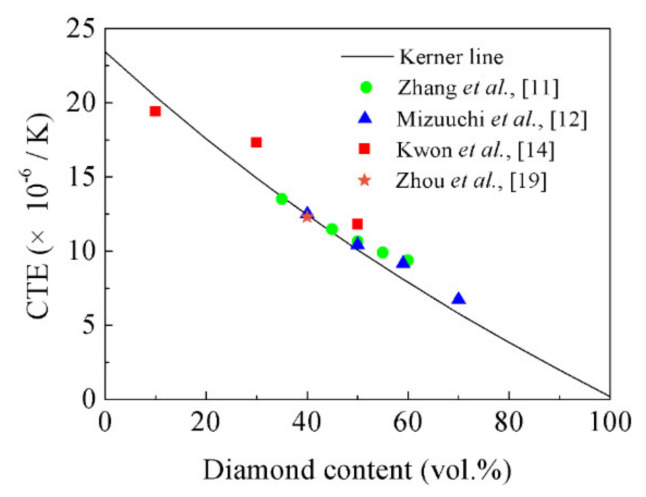
Comparison of the CTE between the references and calculated results.

**Figure 13:**
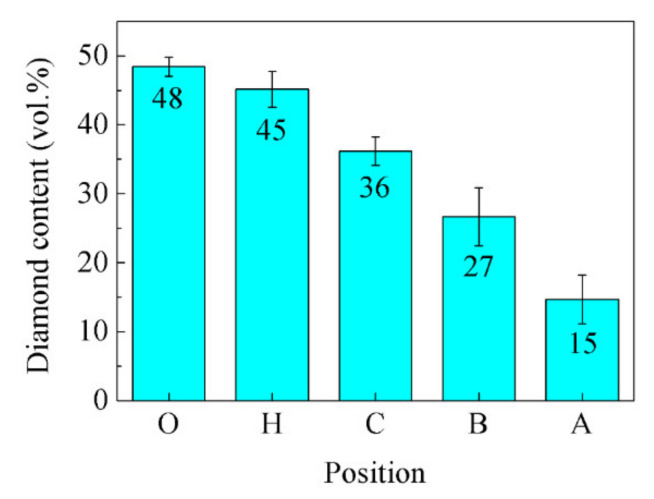
Variation of the diamond content of the diamond/Al graded composites at different positions calculated by the CTE.

Figure 12 shows that the references results of the CTE of the diamond-reinforced metal matrix composites are all in good agreement with the values calculated by the Kerner model. Therefore, the content of diamond particles in the composite can be calculated by the CTE experimental results of the composite, i.e., Equation (1). The content of the diamond particles calculated by the CTE are listed in Table 1. The content of the diamond particles at point “O”, point “H”, point “C”, point “B”, and point “A” are 48.43 vol.%, 45.14 vol.%, 36.18 vol.%, 26.66 vol.%, and 14.69 vol.%, respectively. Figure 13 shows the variation of the diamond content of the diamond/Al graded composites at different positions calculated by the CTE. The content of diamond particles decreases successively along a graded distribution from the bottom of the shell to the top of the shell wall, which is consistent with its density results in Figure 6. The properties of the composites are optimized by FGMs whose microstructure is distributed along the specified direction [24]. Moreover, the relative density is a critical factor in optimizing the mechanical properties and TC of the diamond/Al composites [4,21]. The existing studies have shown that if the diamond surface were coated with W [28], B_4_C [11], and TiC [33], the relative density of the diamond/Al composites can be further increased.

## 4. Conclusions

The independently developed LSS technology has been applied to fabricate near-net shape packaging shells made of diamond/Al composites with graded distributions of the microstructure and properties. It is significant in guiding the industrialization and popularization of these types of materials, such as diamond/Cu composites and diamond/Ag composites. By adjusting the volume fraction of diamond particles in the billet and the size of the LSS chamber, the diamond/Al, Cu or Ag composites with variable diamond content and distribution can be fabricated. Based on the experimental results and analyses, the main conclusions of the present study can be summarized as follows:

1. From the bottom center of the shell to the top of the wall, the contents of the diamond particles are 48 vol.%, 45 vol.%, 36 vol.%, 27 vol.%, and 15 vol.%, respectively. The gradual change in the structure of the packaging shell, reducing the thermal stress and residual stress, helps to improve the thermal stability of the packaging components.
2. The interface of the diamond/Al graded composites presents a mixed bonding state including the diffusion-bonded and partially diffusion-bonded states. The partially diffusion-bonded interface is not enough to form the ligament connecting diamond particles and Al, and reduces the properties of the composites.
3. With the decrease in diamond content, the TC of the composites decreased, while the bending strength and CTE increased. The graded distribution perfectly solves the special requirements at different parts of the packaging shell for performance and helps to improve the thermal stability of packaging components.
4. The sample at the bottom of the shell has the lowest CTE (11.0 × 10^−6^/K) and the highest TC (169 W/m·K); such performance ensures that the CTE of the composites is matched with the CTE of the chip, and dissipates the heat generated by the chip calculation in a timely and effective manner. The sample at the top of the wall of the shell has the highest CTE (19.3 × 10^−6^/K), which is close to the CTE of the metal cover plate, ensuring good weldability.

## Figures and Tables

**Table 1:** Thermophysical property and bending strength of the diamond/Al graded composites at different sampling positions.

Position	Bending Strength (MPa)	TC (W/m·K)	CTE (10^−6^/K)	Diamond Content (vol.%)
O	88.47	169.36	11.0	48.43 ^‡^
H	96.77	161.38	11.8	45.14 ^‡^
C	113.60	150.02	13.8	36.18 ^‡^
B	135.86	135.65	16.2	26.66 ^‡^
A	174.72	108.25	19.3	14.69 ^‡^

^‡^ Calculated by CTE.

**Table 2:** CTE and mechanical parameters of raw materials [32].

Material	CTE (10^−6^/K)	Bulk Modulus (GPa)	Shear Modulus (GPa)
Al	23.5	76	26
Diamond	1.2	442	478

## Data Availability

Data are available on request to the corresponding author.
